# Placement of self-expandable bifurcated metallic stents without use of fluoroscopic and guidewire guidance to palliate central airway lesions

**DOI:** 10.1186/s40248-016-0052-5

**Published:** 2016-04-30

**Authors:** Cengiz Özdemir, Sinem Nedime Sökücü, Levent Karasulu, Seda Tural Önür, Levent Dalar

**Affiliations:** Yedikule Teaching Hospital for Pulmonology and Thoracic Surgery, Zeytinburnu, Istanbul, 34760 Turkey; School of Medicine, Department of Pulmonary Medicine, Istanbul Bilim University, Istanbul, Turkey

**Keywords:** Metallic stents, Bronchoscopy, Central airways

## Abstract

**Background:**

Self-expandable metallic stents (SEMS) can be used to treat malignant obstructions and fistulas of the central airways. SEMS can be placed using different methods. Recently, a rigid bronchoscope has been used for stent placement without the need for fluoroscopy. We retrospectively evaluated patients for whom SEMS were placed using a rigid bronchoscope, without employing guidewires or fluoroscopy. We describe the intra- and post-procedural complications of the method.

**Methods:**

Data collected between January 2014 and July 2015 were retrospectively evaluated by reference to hospital records.

**Results:**

The mean patient age was 58.14 ± 8.48 years (44–72 years) and 13 out of the 14 patients were male. Twelve had lung cancer, one a thyroid papillary carcinoma with a bronchomediastinal fistula, and one an esophageal carcinoma with a tracheoesophageal fistula. Covered metallic Y-shaped stents were placed in all patients. Before placement, argon plasma coagulation was performed on two patients, diode laser treatment on four, and de-obstruction on nine. No procedure-related mortality was noted. Only two patients required follow-up in the intensive care unit; they were moved to a regular ward after two days. No patient required stent replacement or repositioning. The most common early complication was mucus plugs.

**Conclusion:**

Endobronchial placement of covered self-expandable metallic stents was safe and readily performed in patients with airway obstructions. Neither fluoroscopic nor guidewire guidance was required. Neither patients nor staff were exposed to radiation, and costly guidewire guidance was not necessary. The procedure is cost-effective.

## Background

Beside surgical methods, stent applications by endoscopic methods are also used as a part of multidisplinary approach in the treatment of benign or malign central airway lesions [1–3]. Dumon was the first to place silicon stents in the airways. Today, various types of silicon and metallic stents can be used to treat both benign and malignant airway diseases [4].

Although the use of a rigid bronchoscopic approach is essential when placing silicon stents, self-expandable metallic stents (SEMS) can be placed by various methods. The best-known method is flexible bronchoscopy (FB). SEMS can also be placed under fluoroscopic or guidewire guidance in patients under conscious sedation [5, 6]. Various other methods have been described in the literature. The method of choice is influenced by the general clinical condition of the patient, the capacity of the treating unit, and the experience of the team. Guidelines for the use of either guidewires alone or guidewires in combination with fluoroscopy have been published [7, 8]. In one study a technique employing rigid bronchoscopy, without the need for either fluoroscopy or guidewires, has been developed [9].

Metallic stent placement via FB, under fluoroscopic or guidewire guidance, using only local anesthesia, is popular when treating patients for whom surgery and rigid bronchoscopy are contra-indicated. However, under FB guideness fluoroscopic and guidewire guidance is costly, requires specialized technical equipment and experienced staff, and poses a radiation risk [8]. Not all interventional pulmonology units have all the requisite equipment. Electrocautery, argon plasma coagulation (APC), laser treatments, and cryotherapy, are usually needed by patients for whom interventional pulmonology approaches are planned to treat central airway disease. SEMS placement via bronchoscopy under general anesthesia affords many advantages. Bronchoscopy under general anesthesia prevents coughing or patient movement, which render an endoscopic approach difficult. Blood clots and secretions in the airways can be quickly removed. Patency of the stenotic airway segment can be maintained by mechanical dilatation and desobstruction and bleeding control is afforded by pressure exerted by the bronchoscope [10–13].

In this manuscript we review the efficacy and safety of SEMS placement using a rigid bronchoscope, without fluoroscopic or guidewire guidance, in 14 patients with central airway diseases treated in our interventional pulmonology unit.

## Methods

We studied 14 patients with central airway disease in whom SEMS were placed from January 2014 to June 2015. Data were retrospectively collected from patient records. Our local institutional ethics committee approved the work (Yedikule Chest Disease and Thoracic Surgery Training and Research Hospital Ethical Committee; version no:3983-2015/46). All patients were evaluated by both a pulmonologist and a thoracic surgeon before their procedures, and appropriate endoscopic approaches were defined. Clinical status, symptoms, primary diagnosis, prior treatment, and survival were evaluated. SEMS were placed with or without electrocautery and APC or other laser treatment, as appropriate. All procedures were performed using a rigid bronchoscope (Efer Dumon, EFER Endoscopy, La Ciotat, France) under general anaesthesia. Propofol, alfentanil, and rocuronium (a muscle relaxant) were given intravenously. Stent diameter and localization were determined using both rigid and FB (Karl Storz 11001BN1, Berlin, Germany). We placed two different types of commercial SEMS (Micro-Tech, Nanjing, China; Aerstent, Leufen, Germany).

### Intraluminal treatments other than SEMS placement

We used rigid tracheal and bronchial tubes 10–12 mm in outer diameter. To treat intraluminal and mixed-type stenosis evident after devascularization of the endobronchial component of the tumor using a diode laser or APC, a rigid bronchoscope was initially employed to remove mechanical obstructions. If a hemorrhage at the base of the lesion required treatment, APC was used to achieve coagulation. Each extraluminal stenosis was dilated using an esophageal balloon (delivered via an endoscopic balloon dilation catheter; Balton, Warsaw, Poland), or a rigid bronchoscope, prior to stent insertion. A diode laser operating at 980 nm (4–25 W, pulsed mode; Biolitec Ceralas D 25, Jena, Germany) was used for endobronchial treatment. APC (40 W, blended mode/continuous flow) was delivered using a device manufactured by ERBE Elektromedizin Gmbh (Tübingen, Germany). The power levels were those recommended by the manufacturer.

### Stent placement

**Fig. 1:**
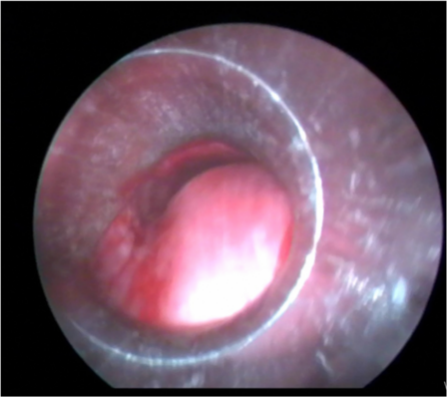
Posterior extrinsic pressure involving the distal part of the trachea and carina which causes 70 % obstruction in the airway lumen. Although this obstruction was dilated with rigid bronchoscopy, enough patency is not maintained and stent application is planned

**Fig. 2:**
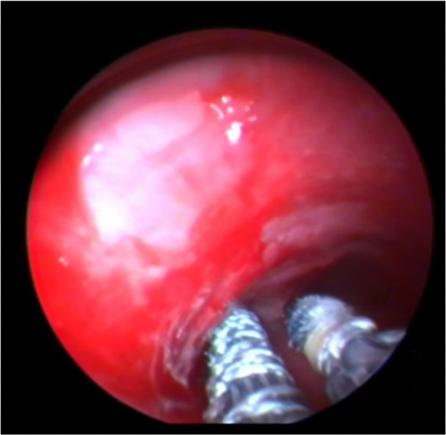
While the patient was being ventilated with the aid of the rigid bronchoscope, stent was moved to the stenotic area with the help of laryngoscope passing through the vocal cords between rigid bronchoscope and trachea wall. Distal to the rigid bronchoscope stent legs were seen to be released

**Fig. 3:**
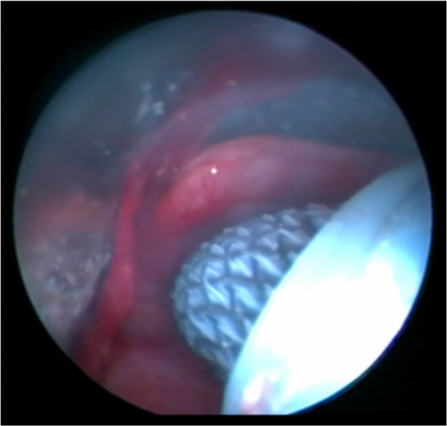
Stent was started to be opened in the airways after the right and left legs of the SEMS were placed. While the right and left legs of the bifurcated stent were pushed forward with slow maneuvers, FB helps for the correct placement of the stent legs in the airway

**Fig. 4:**
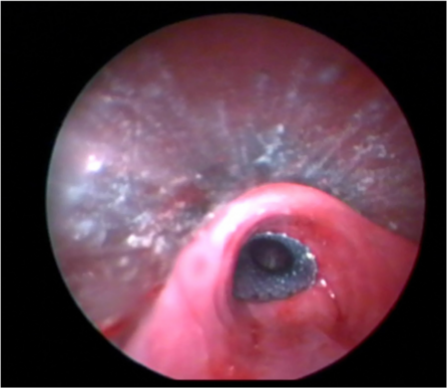
After the correct positioning of the stent, it was rechecked by going inside the stent by FB to check its proximal and distal airway to be sure that any secretions and/or hemorrhage behind the stent were cleared and also to prevent any possible complications (malposition, bronchial wall laceration, hemorrhage, etc.). After the stent was fully opened, extrinsic pressure previously seen on the distal part of trachea and airways was resolved

If airway patency was <50 % after rigid bronchoscopic intervention (dilatation and/or desobstruction), or if we thought it likely that stenosis would soon recur, we placed a stent (Fig. 1). Another indication for stent application was to cover fistula when a fistula stoma was detected by bronchoscopic evaluation in between central airway and esophagus or mediastinum. Before such stent placement, the tube was stabilized at a position proximal to the stenosis after intubation with a rigid 8-mm-outer-diameter tube to allow ventilation. Next, the stent body was passed through the vocal cords to a position anterior to the rigid tube, with the help of the laryngoscope. While the patient was being ventilated with the aid of the rigid bronchoscope, a flexible bronchoscope was passed through the rigid tube. After direct observation of the distal end of the stent via FB, the bronchoscope was moved to the stenotic area and moved forward and backward to visualize the stent meshwork; the bronchial legs of the stent were then directed toward the right and left main bronchi (Figs. 2 and 3). Afterwards, the position of the legs of the stent in the main bronchus was checked by the FB, and when we were sure that the stent was in the correct area, the stent was opened (Fig. 4). Then the position of the stent was rechecked to be sure that stent was opened at the correct location by going inside the stent to check proximal and distal airway of the stent. Finally, any secretions and/or hemorrhage behind the stent were cleared. The recorded procedure time was that from the start of intravenous anaesthesia to extubation. The extent of all endobronchial lesions and appropriate positioning of all stents were confirmed by direct visualization. Fluoroscopy was never used during stent placement.

### Complications

Hemorrhages developing during the procedure were defined as minimal (no additional intervention needed); moderate (controllable by APC and diode laser coagulation); and severe (>100 mL). All hypoxemia (desaturation below 90 % for more than 1 min), any respiratory insufficiency requiring mechanical ventilation, arrhythmia, hypotension, and death within 24 h after the procedure, were considered to be complications. Follow-up FB was performed 24 h after SEMS placement. Airway damage, stent opening, stent migration, and retention of secretions were evaluated.

## Results

The mean patient age (14 patients) was 58.14 ± 8.48 years (44–72 years); two patients were female. SEMS were placed in 12 (85.72 %) patients to treat airway obstructions caused by lung cancer (11 NSCLCs, 1 SCLC); in one (7.14 %) to treat a bronchomediastinal fistula caused by papillary thyroid carcinoma; and in one (7.14 %) to treat a tracheoesophageal fistula caused by esophageal carcinoma. The mean ASA (American Society of Anesthesiologists) patient score prior to intervention was 2.64 ± 0.74 (range 1–4).

Various endoscopic interventions were used during the procedure, alone or in combination. Four patients required diode laser therapy, two APC, and nine mechanical de-obstruction or balloon dilatation. Four patients required SEMS placement only; whereas the others required additional treatments combined with stent placement. In the latter ten patients, SEMS placement was the final procedure. Nine patients received Y-type SEMS 16 × 12 × 12 mm in dimensions; two SEMS of 18 × 14 × 14 mm; and three SEMS of 20 × 14 × 14 mm (Table 1).

**Table 1:** Dermographical characteristics and interventions

Patients’ number, *n*	14
Age	58.14 ± 8.48
Sex (f/m), *n*	2/12
Diagnosis	
Airway obstruction due to lung cancer Bronchomediastinal fistula due to thyroid papillary carcinoma Tracheosophageal fistula due to eosophageal carcinoma	12 1 1
ASA score	2.64 ± 0.74
Diode laser	4
Argon plasma coagulation	2
Balon dilatation or mechanical desobstruction	9
Stents used†	14

ASA score : American Society of Anesthesiologists score

†9 patients 16 × 12 × 12 mm size Y type self expandable metalic stents (SEMS), 2 patients 18 × 14 × 14 mm size Y type SEMS, 3 patients 20 × 14 × 14 mm size Y type SEMS

The mean procedure time was 49.64 ± 13.56 (30–80) min. Two patients developed minimal and four moderate hemorrhage. No severe hemorrhage was observed. No procedure-related mortality occurred. Only two patients were sent to the intensive care unit postoperatively, because of respiratory insufficiency. They underwent non-invasive mechanical ventilation and were moved to a regular ward after 48 h. Airway status and stent function were evaluated in all patients 24 h after their procedures. No stent replacement or repositioning was required. Three patients exhibited retention of secretions in the absence of severe stent obstruction. The secretions were cleared via FB.

## Discussion

We used rigid bronchoscopy and FB to place bifurcated SEMS. The procedure was efficient and safe. Neither fluoroscopy nor guidewires were needed to treat central airway obstructions caused principally by cancerous lesions. No severe complications were observed and all stents were successfully placed. Today, both silastic and metallic stents can be used to treat central airway lesions. Bifurcated stents are preferred when airway stenosis involves the carinal area, or when a tracheoesophageal fistula is present or a fistula might develop at the anastomotic site after operation. Rigid bronchoscopy must be used for stent placement in such situations [14–16]. In addition, team experience is important, and various maneuvers are required to prevent laceration of the airways, main vessels, and tracheobronchial wall when placing silicon stents [17].

Modern SEMS are mostly silicon-covered, made of nickel titanium alloy, and feature shape memory. SEMS placement can be achieved by rigid bronchoscopy under general anaesthesia or employing fluoroscopic or guidewire guidance under local anaesthesia. Most commonly, FB is used under local anaesthesia with fluoroscopic guidance [6, 7]. Herth et al. placed plain metallic stents in 96 patients using FOB with guidewire guidance; fluoroscopy was not employed. No procedure-related complication was noted [8]. Lin et al. used FB with guidewire guidance to treat airway lesions; fluoroscopy was not employed. Plain metallic stents were placed in 26 patients who then underwent endotracheal tube intubation because of acute respiratory insufficiency. Later, 14 patients were weaned from mechanical ventilation [18]. The cited study did not describe procedure-related complications in detail; but one case of pneumothorax and one of stent migration were reported.

SEMS placement without the use of either fluoroscopic or guidewire guidance has been reported in one study [9]. Husain et al. placed ultraflex airway stents in 66 patients with malignant and benign airway obstructions. Four complications were reported < 12 h postoperatively; none of which were stent-related. These early complications were hypoxia, lobular collapse, hemorrhage, and respiratory insufficiency. Differently, in our study, bifurcated stents were placed in all patients. We observed minimal or moderate hemorrhage in six patients; all hemorrhages were controlled. We encountered no complication-related deaths, but two patients required non-invasive mechanical ventilation for two days after their procedures. All bifurcated stents were successfully placed and no stent-related complication was observed.

Various endoscopic methods can be used, alone or in combination, to treat central airway lesions, depending on the underlying disease, the type of stenosis, and the clinical condition of the patient [19, 20]. In patients for whom endoscopy is planned using a multidisciplinary approach, and who can tolerate general anaesthesia, rigid rather than FB affords advantages [21]. The borders of the stenotic segment can be directly evaluated and dilatation, hot intervention, and mechanical de-obstruction, can be performed. Hemorrhage and ventilatory deficiency can be effectively managed, and secretions aspirated. We prefer to add various interventions, via rigid bronchoscopy, during SEMS placement in our interventional pulmonology unit. We combined such interventions with SEMS placement in ten patients (71.4 %).

Although complications may be of concern, and although general anaesthesia is costly, safety considerations suggest that rigid bronchoscopy is the best option for endoscopic interventions, and this is the practice in our unit.

General anaesthesia increases procedure time and is associated with anaesthesia-related complications. Our mean procedure time was 49.64 ± 13.56 (30–80) min. When FB was used to place SEMS (ultraflex stent) in sedated patients, the mean procedure time was 24.2 ± 8.8 min [18]. The longer time of our study is explained by the fact that we combined stent placement with other procedures. Using FOB, a stent is placed without prior de-obstruction; rigid bronchoscopy allows obstructions to be removed. The fact that we did not observe any procedure-related complication associated with procedure duration supports the notion that our method is safe.

We studied short-term, but not long-term, effectiveness and safety. Upon FB performed 24 h postoperatively, three patients exhibited secretion retention that did not severely obstruct the stents; therefore the secretions were removed. No stent malpositioning or migration was observed.

The limitations of our study is that we did not evaluate the long term results of the stent application but only the application method. Another limitation is that our study population is small.

## Conclusion

In conclusion, endobronchial placement of covered self-expandable bifurcated metallic stents via rigid bronchoscopy appears to be safe and simple in patients with airway obstructions and neither fluoroscopic nor guidewire guidance is crucial. Also by rigid bronchosopy, treatment modalities other then stent placement can be applied. This approach eliminates exposure of patients and staff to radiation.
